# Anti-tumor effect of adenovirus-mediated gene transfer of pigment epithelium-derived factor on mouse B16-F10 melanoma

**DOI:** 10.1186/1756-9966-28-75

**Published:** 2009-06-05

**Authors:** Li-Ping Yang, Ping Cheng, Xing-Chen Peng, Hua-Shan Shi, Wei-Hong He, Feng-Yu Cui, Shun-Tao Luo, Yu-Quan Wei, Li Yang

**Affiliations:** 1 State Key Laboratory of Biotherapy and Cancer Center, West China Hospital, West China Clinical Medicine School, Sichuan University, Chengdu, Sichuan, PR China; 2 School of Life Science, Sichuan University, Chengdu, Sichuan, PR China

## Abstract

**Background:**

Angiogenesis plays an important role in tumor growth, invasion, and eventually metastasis. Antiangiogenic strategies have been proven to be a promising approach for clinical therapy for a variety of tumors. As a potent inhibitor of tumor angiogenesis, pigment epithelium-derived factor (PEDF) has recently been studied and used as an anticancer agent in several tumor models.

**Methods:**

A recombined adenovirus carrying PEDF gene (Ad-PEDF) was prepared, and its expression by infected cells and in treated animals was confirmed with Western blotting and ELISA, respectively. Its activity for inhibiting human umbilical vein endothelial cell (HUVEC) proliferation was tested using the MTT assay. C57BL/6 mice bearing B16-F10 melanoma were treated with i.v. administration of 5 × 10^8 ^IU/mouse Ad-PEDF, or 5 × 10^8 ^IU/mouse Ad-Null, or normal saline (NS), every 3 days for a total of 4 times. Tumor volume and survival time were recorded. TUNEL, CD31 and H&E stainings of tumor tissue were conducted to examine apoptosis, microvessel density and histological morphology changes. Antiangiogenesis was determined by the alginate-encapsulated tumor cell assay.

**Results:**

The recombinant PEDF adenovirus is able to transfer the PEDF gene to infected cells and successfully produce secretory PEDF protein, which exhibits potent inhibitory effects on HUVEC proliferation. Through inhibiting angiogenesis, reducing MVD and increasing apoptosis, Ad-PEDF treatment reduced tumor volume and prolonged survival times of mouse bearing B16-F10 melanoma.

**Conclusion:**

Our data indicate that Ad-PEDF may provide an effective approach to inhibit mouse B16-F10 melanoma growth.

## Introduction

Melanoma is a malignant tumor derived from melanocytes which are found predominantly in skin but also in the bowel and the eye. Approximately 160,000 new cases of melanoma are diagnosed and around 48,000 melanoma-related deaths occur worldwide each year [1]. Despite many years of intensive research, surgical resection and systemic chemotherapy are still the main therapeutic strategies for malignant melanoma. Unfortunately, for advanced melanoma, surgical resection is insufficiently effective while chemotherapy introduces significant side effects [2]. Compared to other type of skin cancer, melanoma is more rare but often associated with a high mortality, accounting for 75% of all deaths from skin cancer [3]. To explore new therapeutic agents/methods with less side effects is a major initiative in melanoma research.

One hallmark of melanoma progression is angiogenesis, which is induced by angiogenic factors released by tumor cells and characterized as the formation of a new vascular network from pre-existing blood vessels. Angiogenesis facilitates tumor growth by supplying nutrients and oxygen, while promoting tumor invasion and metastases [4]. Antiangiogenesis has been proposed as a therapeutic strategy for cancer treatment since the 1970s, but it has been limited by the unavailability of antiangiogenic agents and/or inefficient administration methods. In the past two decades, several antiangiogenic factors, such as angiostatin, endostatin, thrombospondin and pigment epithelium-derived factor (PEDF), have been found and characterized [5]. As a new family of anti-tumor agent candidates, they are under active investigation by many researchers. Accumulating data show antiangiogenic agents have promising efficacy in tumor treatment [6]. Because PEDF selectively and potently suppresses new vessel growth with least impact on pre-existing vessels, it is one of the top candidates for tumor therapy [5].

PEDF is a 50 kDa glycoprotein that was first purified from the conditioned medium of cultured primary human fetal retinal pigment epithelial cells [7]. Later, it was found that PEDF is widely expressed in human tissues, including the adult brain, spinal cord, plasma, liver, bone, eye, heart, and lung [5]. PEDF is a multi-functional serpin family protein. It has been reported that it activates the Fas/FasL death pathway and subsequently induces endothelial cell death, and also regulates the balance between proangiogenic and antiangiogenic factors [8]. One prominent feature of PEDF is the selective inhibition of neovascularization, which is extremely important to minimize the side effects in tumor treatment. The underlying mechanism is still not well understood, but it has prompted scientists to apply it in cancer treatment in a variety of forms including purified, recombinant, PEDF peptide 327 to 343, and gene transfer [9]. Adenovirus is the widely utilized gene transfer vehicle in a variety of gene therapies; however, adenovirus-mediated gene transfer of PEDF for tumor treatment is rarely reported.

In this study, we constructed a recombinant PEDF-expressing adenovirus (Ad-PEDF) and tested its anti-tumor efficacy in a mouse B16-F10 melanoma model. Our data indicate that the Ad-PEDF treatment of melanoma-bearing mice results in an increase of serum PEDF and reduction of tumor angiogenesis, growth, and animal death. The adenovirus-mediated gene transfer of PEDF is thus a promising therapeutic strategy for melanoma and other angiogenic tumors.

## Methods

### Recombinant adenovirus construction and viral preparation

According to the cDNA sequence of PEDF in genebank, we designed a pair of PEDF primers that contain a Pme I restriction site (underlined in the following) in both primers (5'-AGCTTTGTTTAAACATGCAGGCCCTGGTGCTACTCCTC-3' and 5'-AGCTTTGTTTAAACTTAGGGGCCCCTGGGGTCCAGAATC-3'). Using these primers, we amplified human PEDF cDNA with RT-PCR. PCR product was digested with Pme I and its sequence was confirmed. Using AdEasy system, we first clone PEDF cDNA into a shuttle vector pAdenoVator-CMV5 at Pme I and Bam H I site, in which PEDF expression is under the control of the constitutive cytomegalovirus (CMV) promoter. The recombinant shuttle plasmid was used to rescue the replication-defective adenovirus [10]. Ad-luciferase and Ad-Null was prepared as the construction of Ad-PEDF, except luciferase gene or no objective gene was inserted.

The viral particles were amplified in human embryonic kidney (HEK293) cells (ATCC Rockville Maryland, USA), which were maintained in DMEM medium (Gibico BRL, Grand Island, New York, USA) with 10% fetal bovine serum (FBS) plus 100 μg/ml amikacin in a 37°C humidified chamber with 5% CO_2 _atmosphere. The harvested viral particles from the cultures were purified by double cesium chloride (CsCl) gradient ultracentrifugation followed by dialysis. Final aliquots of virus were measured by absorption (A260). The virus titer was quantified using a standard TCID50 assay.

### Cell culture and adenovirus infection

Primary human umbilical vein endothelial cells (HUVECs) were collected and cultured as previously described [11]. The CT26 mouse colon carcinoma and B16-F10 mouse melanoma cell lines were purchased from the American Type Culture Collection (ATCC, Rockville Maryland, USA) and cultured in RM1640 medium (Gibico BRL, Grand Island, New York, USA) supplemented with 10% FBS and 100 μg/ml amikacin. 2.5 × 10^5 ^CT26 or B16-F10 cells were plated into 6-well plates and grew to 70%~80% confluence. The cells were washed three times gently by serum-free medium and infected with Ad-PEDF or Ad-null (both at MOI50, 2.5 × 10^7 ^pfu per 5 × 10^5 ^cells) in 0.5 ml serum-free medium, with normal saline as the non-infection control. After incubation for 90 minutes at 37°C, 1.5 mL complete medium with 10% FBS was added to wells. Supernatants were collected after further culture for 72 hours and stored at -80°C for further analysis.

### Western blotting analysis

Western blot analysis was performed as described previously [12]. Briefly, the supernatant was concentrated by super filter (5 kDa, Minipore) and mixed with an equal volume of sodium dodecyl sulfate (SDS) sample buffer. The proteins were separated by SDS-polyacrylamide gel electrophoresis (PAGE) and electronically transferred onto a polyvinylidene difluoride membrane (PVDF, Bio-Rad, Richmond, CA, USA). The blots were probed with a mouse anti-human PEDF monoclonal antibody (3:1000, mAb; R&D Systems, Boston, Massachusetts, USA) plus a peroxidase-conjugated secondary antibody, goat anti-mouse IgG (1:10,000, ZSGB-BIO, Beijing, China). The protein bands were visualized using an enhanced chemiluminescence (ECL) detection system (Pierce, Rockford, Illinois, USA).

### 3-(4,5-dimethylthiazol-2-yl)-2,5-diphenyl tetrazolium bromide (MTT) colorimetric assay

The MTT Assay was used to determine the effect of PEDF derived from Ad-PEDF infected cells on human umbilical vein endothelial cells (HUVECs). Three types of supernatants from B16-F10 cells infected with Ad-PEDF, Ad-Null or NS, respectively, were prepared as described above. Each type of supernatant was further diluted into a series of 1/2 dilutions in six tubes (from 1:2 to 1:64). Each supernatant dilution was added into triplicate wells (50 μl/well) of HUVECs which were seeded on 96-well plates on the previous day (2 × 10^3 ^cells in 50 μl complete medium per well). The cells were incubated at 37°C in 5% CO_2 _for 72 hours. Then, each well received 10 μl MTT solution (5 mg/mL). After a 4-hour incubation, the media was removed and 150 μl dimethyl sulfoxide was added. After 20 min of incubation, the OD value was determined by a microplate reader (3550-UV, BIO-RAD, USA)[13]. The following formula was used to calculate the inhibition rate of HUVEC proliferation: [1 - (experimental group OD value - negative control OD value)/(positive control OD value - negative control OD value)] × 100%.

### Animal experiments

Six to 8 week-old female C57BL/6 and BALB/c mice were purchased from the West China Experimental Animal Center of Sichuan University (Sichuan, China). Mice were permitted 1 week to acclimate to their environment before manipulation. All surgical procedures were completed in accordance with the guidelines on the care and use of laboratory animals for research purposes by the West China Hospital Cancer Center's Animal Care and Use Committee. C57BL/6 mice were inoculated with 1 × 10^5 ^B16-F10 melanoma cells s.c. in the right flank. Primary tumors usually became palpable on day 7–8 and with an average diameter of 3 mm. On day 9, the tumor-bearing mice were randomly assigned into 3 groups and each group contained 8 mice. Each mouse in Ad-PEDF group received 5 × 10^8 ^IU Ad-PEDF virus in 0.1 ml via i.v. injection on day 9, 12, 15, and 18 with a total of 4 times. The mice in the control groups received 5 × 10^8 ^IU Ad-Null or normal saline (NS), serving as vector and injection control, respectively. The details of the treatment were described previously [14]. Tumor dimensions were measured with calipers on day 9, 12, 15, 18, 21 and 24 with a total of 6 times. The tumor volumes were calculated according to the following formula: length × width^2 ^× 0.52. Two mice from each group were bled to collect serum on day 22, which was used to examine the PEDF concentration in serum. Surviving mice in Ad-PEDF groups were monitored up to 42 days; all other mice become moribund by day 24 and were sacrificed. Subcutaneous tumors from sacrificed mice were removed and fixed in 4% formaldehyde solution for immunochemistry staining and histological analysis.

### Detection of PEDF concentration in serum

Concentrations of PEDF in serum were determined using a commercial PEDF ELISA kit (ADL, Biotech. Dev. Co., USA) following the manufacturer's instructions. Briefly, 50 μl serum and 50 μl PEDF monoclonal antibody were added to every well of the pre-coated ELISA plate and the plate was incubated at 37°C for 1 hour. After wash, 80 μl of streptavidin-HRP was added and incubated at 37°C for 30 minutes. After wash, 50 μl substrate A and B was added, respectively, and incubated for 10 minutes at 37°C, followed by 50 μl stop solution. The absorbance was read immediately at 450 nm in a spectrophotometer [15]. There were 2 serum samples in each group, and each sample were applied to 3 replicated wells.

### Luciferase assay for virus distribution

Virus distribution was analyzed using the luciferase reporting system, as reported previously [16]. C57BL/6 mice were inoculated with 1 × 10^5 ^B16-F10 melanoma cells s.c. in the right flank. On day 9, the tumor-bearing mice were randomly assigned into 2 groups and each group contained 3 mice. Experimental group received 5 × 10^10 ^IU Ad-luciferase and control group received 5 × 10^10 ^IU Ad-null virus in 0.1 ml via i.v. injection. Seven days later, the mice were sacrificed. Heart, liver, spleen, lung, kidney and tumor from each mouse were collected and individually stored in liquid nitrogen. Using a luciferase assay system kit (Promega, Madison WI, USA), we analyzed luciferase expression in each type of collected organ, according to the manufacturer's instructions. Briefly, the same organs from the same group were pooled and ground to a fine powder in a mortar containing liquid nitrogen. The fine powder was dissolved and further processed in CCLB solution in the assay kit. The resultant supernatants were collected and subjected to determination of relative light units (RLU, synergy 2, Biotek, Germany), along with a group of standard samples in the kit. The amount of luciferase in each sample was calculated on the basis of the standard curve.

### Detection of apoptosis and microvessel density (MVD)

On day 24 after mouse inoculation with melanoma cells, subcutaneous tumors from Ad-PEDF, Ad-null and NS treated mice were collected, fixed, embedded in paraffin, and cut into 3–5 μm sections. The apoptotic cells within the tumor tissue were evaluated using the DeadEnd Colorimetric Terminal Deoxynucleotidyl Transferase-Mediated dUTP Nick-End Labeling (TUNEL) System (Promega, Corporation, Madison, Wisconsin, USA) following the manufacturer's protocol. Ten high power fields on each slide and three slides from each animal were examined. Apoptosis index was calculated by dividing the number of apoptotic cells by the total number of cells in the field.

The method reported by Weidner et al was adopted to quantify MVD in tumor tissues [17]. Briefly, 5 μm tumor sections were stained for the epithelial cell marker, CD31. The procedure of immunostaining for CD31 was previously described in detail [18]. The following antibodies and reagents were used: goat anti-mouse CD31 mAb (1:200, Santa Cruz Biotechnology, Santa Cruz, California, USA), biotinylated polyclonal rabbit anti-goat (1:100, Santa Cruz Biotechnology, Santa Cruz, California, USA), ABC kit (Boster biological engineering company, Wuhan, China) and DAB visualization system (ZSJQ Biotechnology, Beijing, China). The resultant sections were first examined at low magnifications (×40 and ×100) to identify the vascular-rich area in the tumor. Within this area, the CD31-positive microvessels were counted in a single high-power (×200) field. Any CD31 stained single or cluster of cells was considered a single countable microvessel. Adjacent sections were stained with hematoxylin and eosin (H&E) and examined for tissue structure and histological morphology. Each group contains 2 mice, and 3 sections from each mouse.

### Alginate-encapsulated tumor cell assay

The alginate-encapsulated tumor cell assay was used to measure tumor angiogenesis *in vivo*, as previously described [14,18]. Briefly, B16-F10 or CT26 cells in 1.5% (m/v) sodium alginate solution (Sigma-Aldrich, St. Louis, Missouri, USA) was dropped into a swirling 0.25 M CaCl_2 _solution to prepare alginate beads (1 × 10^5 ^cells/bead). Four resultant beads were implanted s.c. on both dorsal sides of BALB/c female mice (2 beads/side). On the next day, mice were randomly assigned into 3 groups (n = 2), and each group was i.v. injected with 0.1 ml Ad-PEDF (5 × 10^8^IU/mouse), Ad-Null (5 × 10^8 ^IU/mouse), or NS, respectively. After a week, this same treatment on each mouse was repeated. On day 11 after tumor cell implantation, all mice were injected i.v. with 100 μl FITC-dextran (Sigma-Aldrich, St. Louis, Missouri, US) solution (100 mg/ml), which is a plasma-borne tracer extravasating into tissue interstitial fluid from plasma within 20 minutes. Alginate beads were exposed surgically and photographed with a digital camera (model, Canon, Japan). Then, the beads were removed and vortexed in a tube containing 2 ml NS. After centrifugation, the supernatant was collected and subjected to a fluorescence spectrophotometer for the measurement of fluorescence intensity. The amount of FITC-dextran was calculated and used to estimate the amount of blood supply and angiogenesis status.

### Statistical analysis

SPSS program (version 15.0, SPSS Inc., USA) was used for statistical analysis. Log-rank test was used to compare survival rate among groups. ANOVA was used to determine statistical significances in remaining comparisons in this study. The difference is considered as significant if *p *< 0.05.

## Results

### Recombinant Ad-PEDF virus successfully transferred PEDF gene into tumor cells and produced secretory PEDF protein in vitro

**Figure 1:**
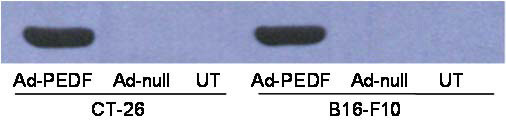
**Expression of human PEDF in Ad-PEDF infected cell lines**. Supernatant from Ad-PEDF, Ad-Null infected and normal saline (NS) treated CT26 and B16-F10 cells were collected and subjected to Western blot analysis with an anti-human PEDF mAb. Human PEDF was detected as a single band of 50 KDa in Ad-PEDF infected cells, but neither in Ad-null infected nor NS-treated cells.

Whether an adenovirus-mediated gene transfer is successful or not mainly depends on its capacity to infect host cells and express the recombinant gene. Therefore, we first tested whether our recombinant Ad-PEDF virus is capable of infecting cells and expresses PEDF protein *in vitro*. CT26 and B16-F10 cell lines were infected with Ad-PEDF, Ad-null or treated with normal saline (NS). Three types of supernatant from each cell line were prepared and subjected to Western blotting analysis. As shown in Fig. 1, PEDF was detected in supernatant from both cell lines infected by Ad-PEDF virus, but neither in Ad-null infected nor NS treated cells. These results indicate that our recombinant adenovirus successfully transfers the PEDF gene into cultured cells and produces secretory protein.

### PEDF protein from Ad-PEDF infected cells exhibited a potent inhibitory effect on HUVEC proliferation

**Figure 2:**
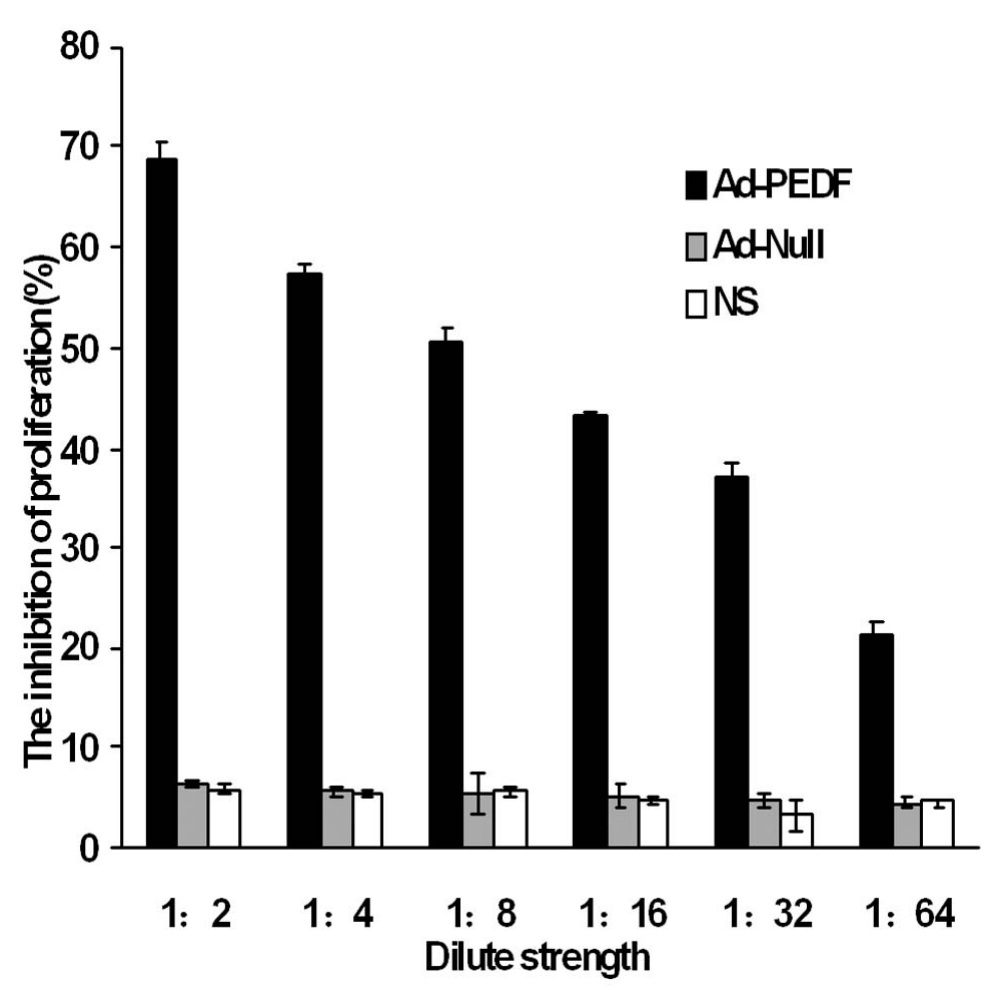
**Inhibitory effect of recombinant PEDF on HUVEC proliferation *in vitro***. The culture supernatants were collected from Ad-PEDF, Ad-null infected and NS treated B16-F10 cells. A 1:2 dilution series of each supernatant were further prepared and applied to HUVEC cells. The proliferation of HUVEC was measured with an MTT assay. The supernatant from Ad-PEDF infected cells inhibited the proliferation of HUVEC in a dose-dependent manner.

Next, we tested whether Ad-PEDF from infected cell possess inhibitory bioactivity on the proliferation of epithelial cells. Using the MTT assay, we measured HUVEC cell proliferation and viability after treatment of supernatant from Ad-PEDF infected B16-F10 cells or control supernatant. As shown in Fig 2, supernatant from Ad-PEDF infected B16-F10 cells significantly inhibited the proliferation of HUVEC with an inhibitory rate of 68.8% ± 1.5% at a 1:2 dilution. Furthermore, this inhibitory rate declines with the increase of dilution, suggesting a dose-dependent effect. In contrast, the control supernatant from Ad-Null infected or NS treated B16-F10 cells had no effect on HUVEC proliferation, which did not change with the dilution. These results indicate that secretory PEDF is functional and capable of mediating a potent inhibitory effect on HUVEC proliferation.

### Ad-PEDF treatment inhibited tumor growth in vivo and prolonged the survival time of the tumor-bearing mice

**Figure 3:**
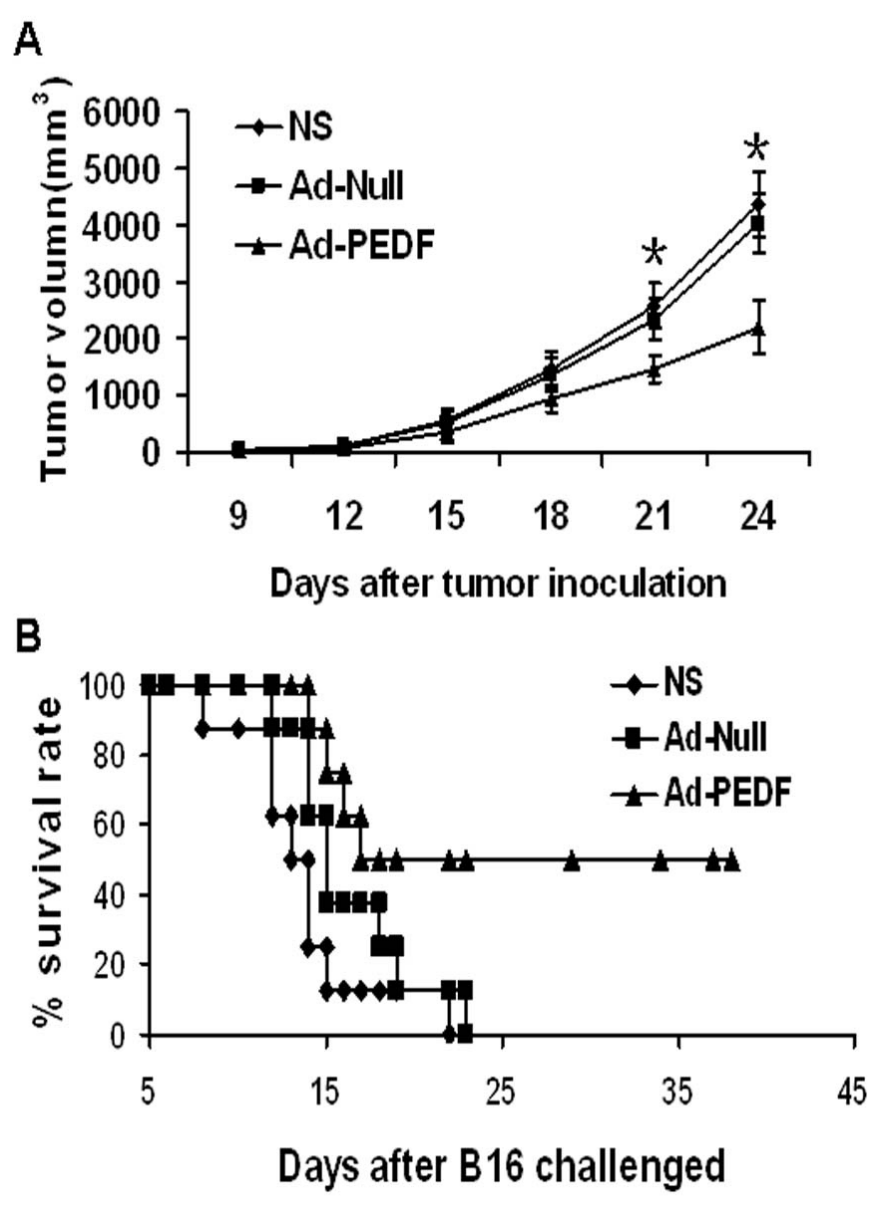
**Anti-tumor efficacy of Ad-PEDF *in vivo***. Three groups of C57BL/6 mice bearing B16-F10 melanoma were treated with NS or 5 × 10^8 ^IU Ad-PEDF or Ad-Null at day 9, 12, 15, 18 and 21 after inoculation, respectively. Tumor sizes on each mouse were measured every 3 days and survival in each group was monitored daily. A. Significant differences were found in tumor volume (*p *< 0.05) between Ad-PEDF treated and the control groups. B. Significant increase of survival rate and prolonged survival times were observed in Ad-PEDF treated mice (log-rank test, *, *p *< 0.05, vs controls). n = 8.

After confirmation of the success for PEDF gene transfer and expression of functional PEDF protein *in vitro*, we examined the anti-tumor efficacy of Ad-PEDF treatment in a mouse tumor model. As shown in Fig 3A, from day 21 after tumor cell inoculation, the tumor volume in Ad-PEDF treated mice started to show significant differences from those in controls (*p *< 0.05). Tumor volumes in the Ad-PEDF treated group was 1447.8 ± 244.4 mm^3^, in contrast to 2337.4 ± 365.8 mm^3 ^in Ad-Null group and 2578.2 ± 406.7 mm^3 ^in NS group on day 21. On day 24, the tumor size in Ad-PEDF, Ad-null and NS groups were 2195.1 ± 462.9 mm^3^, 4013.3 ± 518.3 mm^3^, and 4361.3 ± 569.6 mm^3^, respectively. The time of mouse death was recorded and used to calculate the survival rate. As shown in Fig 3B, the NS treated group showed 50% survival at day 13 and 0% on day 23, and the Ad-null group showed 50% survival at day 14 and 0% on day 24. In contrast, Ad-PEDF group had a 50% survival rate at day 38 and persisted up to day 42. Log-rank test indicated that survival rate in Ad-PEDF group is significantly higher than in control groups (p < 0.05)

### Ad-PEDF treatment increased serum PEDF and adenovirus mainly distributed in liver

**Figure 4:**
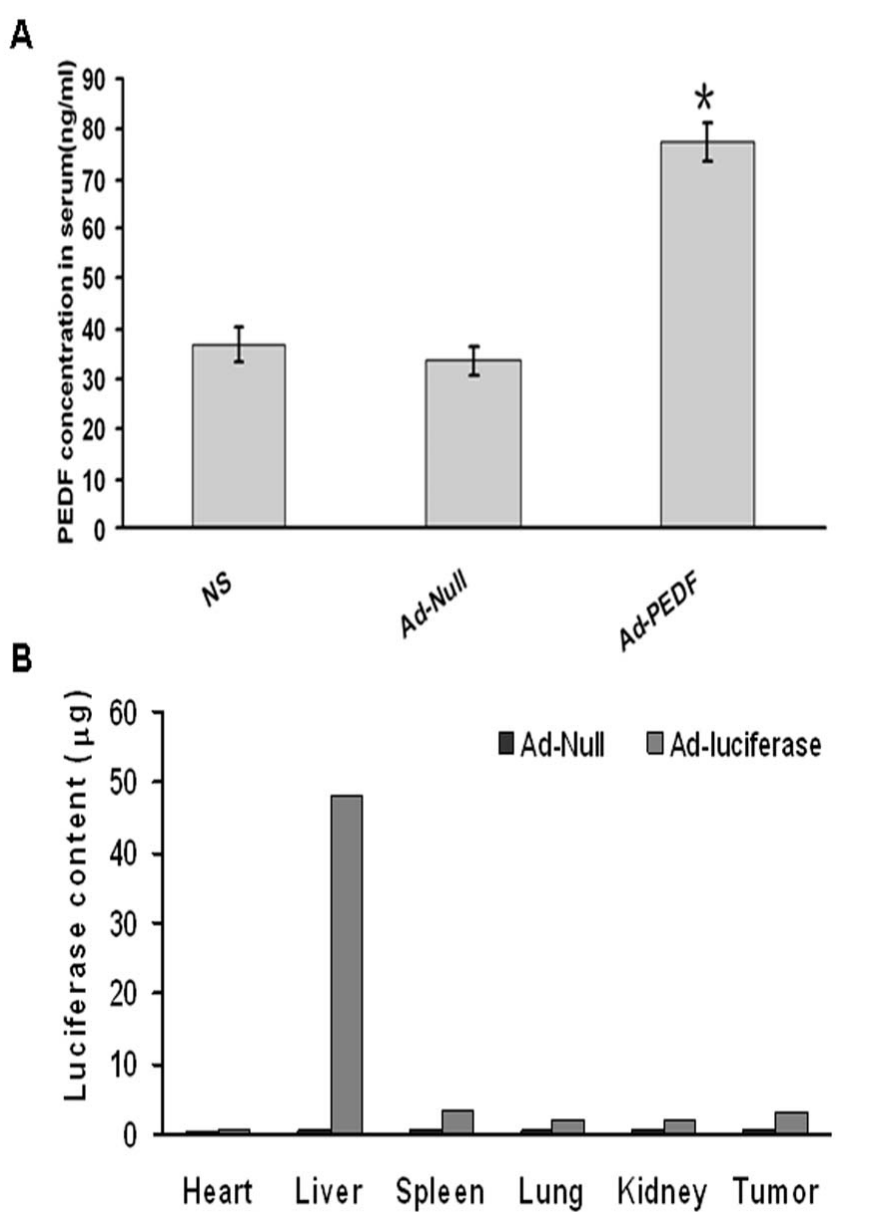
**Serum PEDF and viral distribution in mice after Ad-PEDF treatment**. A. Serum collected from mice bearing B16-F10 melanoma on day 22 after tumor inoculation was processed and subjected to an ELISA analysis to measure PEDF concentration. Compared to Ad-null or NS treated mice, serum PEDF concentration significantly increased in mice treated with Ad-PEDF (ANOVA, *, *p *< 0.05). B. The distribution of i.v. injected virus. The luciferase content represents the amount of virus. n = 2.

To assess whether the above observed smaller tumor volume and increased survival rate in Ad-PEDF group is associated with the effect of PEDF, we measured PEDF concentration in the serum. As shown in Fig 4A, on day 22 after tumor cell inoculation, PEDF level in Ad-PEDF group was significantly higher than control groups, 77.36 ± 3.78 ng/ml vs 33.62 ± 2.79 ng/ml in Ad-null and 36.87 ± 3.35 ng/ml in NS groups, respectively (p < 0.05). This result indicates that Ad-PEDF successfully transferred PEDF to mice and produced secretory PEDF proteins.

Next, we determined the source of PEDF by analyzing the distribution of i.v. injected virus. As shown in Fig 4B, using the luciferase reporting system, we found that the viruses mainly distributed in the liver, in agreement with many adenovirus infection models. This result suggests that while Ad-PEDF infected multiple organs, including the tumor, the liver is the major organ that adenovirus targeted and likely is the significant source of the serum PEDF.

### Ad-PEDF treatment increased apoptosis and decreased MVD in tumor tissue

**Figure 5:**
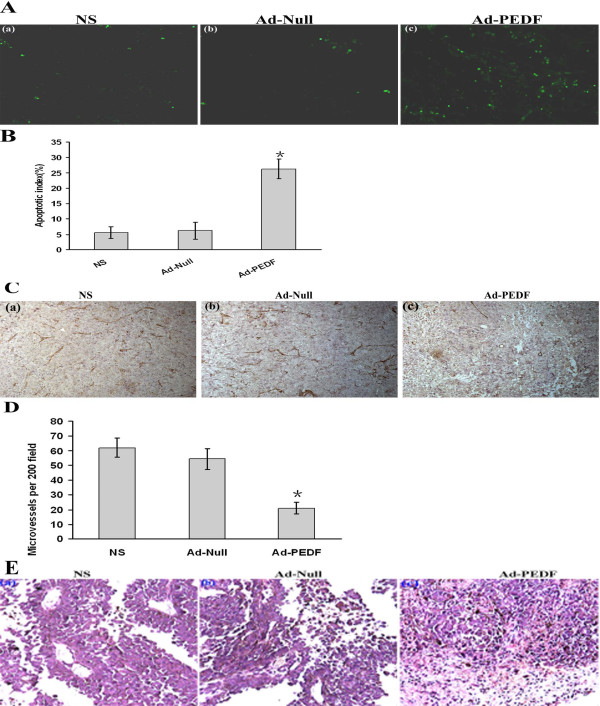
**TUNEL, CD31 and histological staining for tumor tissue**. On day 24 following inoculation, tumor tissue from tumor-bearing mice treated with NS (a), Ad-Null (b), or Ad-PEDF (c) were sectioned and stained with FITC-dUTP, CD31 mAb or H&E. A. Apoptotic cells (green) were identified by TUNEL and examined under a fluorescence microscope (Original magnification, ×200). B. ANOVA analysis detected significant differences in the apoptotic index between Ad-PEDF group and control groups (*p *< 0.05). C. Micrographs show tumor tissue sections stained with anti-CD31 antibody (Original magnification, × 200) after NS, Ad-Null or Ad-PEDF treatment. D. Quantitative results for microvessel density (MVD) in tumor tissue. Ad-PEDF group shows a significant decrease of MVD compared to control groups (*p *< 0.05). E. Micrographs show tumor tissue sections stained with H&E. Decreased density of vessels and noticeable necrosis was observed in tumors from Ad-PEDF treated mice (c). In contrast, tumor cells grew well with less necrosis in NS (a) or Ad-Null group (b). (Original magnification, ×400). n = 2; 3 sections/mouse.

In the proceeding experiments, we observed the reduced tumor volume and increased serum PEDF after Ad-PEDF treatment, in comparison to control, however, the majority of the virus was entrapped in liver and did not target the tumor tissue. It is important to demonstrate whether serum PEDF indeed acts on tumor tissue and causes histological change. To address this question, we determined apoptosis in tumor tissue after Ad-PEDF treatment using TUNEL staining. As shown in Fig 5A, within a similar field of view, may more apoptotic cells (with green nuclei) in tumor tissues were observed in Ad-PEDF treated mice than in Ad-null or NS treated mice. For the quantitative comparison, the apoptosis index in each group was calculated. The apoptosis index was significantly higher in Ad-PEDF group than in Ad-Null and NS groups with values of 26.3% ± 3.3% *v.s. *6.3% ± 4.7% and 5.6% ± 1.9%, respectively (p < 0.05, Fig 5B). These data suggest that decreased tumor volumes after Ad-PEDF may be caused by increased apoptosis.

To further determine whether the increase in apoptosis of Ad-PEDF treated tumor tissue was associated with the antiangiogenic effect of PEDF, we analyzed MVD of tumor tissues in each group. As shown in Fig 5C, intensive CD31 immunoreactive microvessels was observed in tumor tissue from mice treated by NS and Ad-null, but only moderate CD31 staining present in tumor tissue from mice treated by Ad-PEDF. For comparison, CD31-positive single or a cluster of cells were counted as the microvessels, and MVD was calculated for each group with the formula described in the materials and methods. MVD of tumor tissues from Ad-PEDF treated mice exhibited a significant decrease than from Ad-null or NS treated mice, (21 ± 4, 54.3 ± 7.2, 62 ± 6.5, respectively) (p < 0.05, Fig 5D). These data suggest that the decreased angiogenesis after Ad-PEDF treatment may be responsible for the increased apoptosis.

Adjacent sections were stained with H&E to evaluate the morphologic changes after Ad-PEDF or control treatments. Consistent with the results of CD31 immunochemistry staining, less vessels and remarkable necrosis areas were observed in tumor tissue from Ad-PEDF treated mice in comparison to Ad-null or NS treated mice (Fig 5E). Collectively, these data suggest that serum PEDF from infected host cells is sufficient to inhibit tumor angiogenesis, subsequently promote apoptosis, reduce tumor progression and prolong survival time.

### Ad-PEDF treatment inhibited the development of tumor angiogenesis

**Figure 6:**
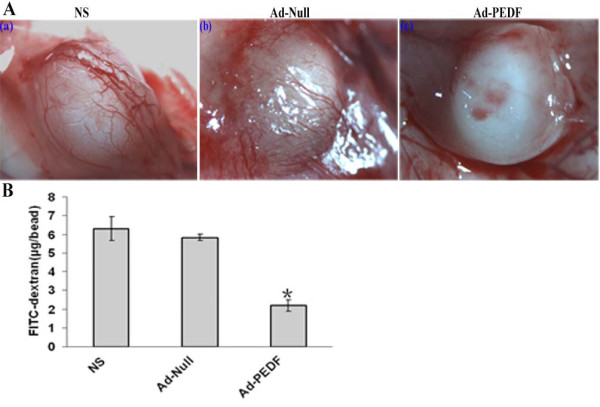
**Alginate-encapsulated tumor cell assay for the inhibition of angiogenesis**. Mice bearing alginate beads containing CT26 tumor cells were treated with NS, Ad-Null, or Ad-PEDF twice on day 1 and 8, respectively. On day 11, all mice received an injection of FITC-dextran and were sacrificed 20 min later. A. Photographs show surface of alginate beads from different groups. B. FITC-dextran uptake in the tumor tissue was significantly decreased in mice treated with Ad-PEDF compared to mice treated with NS (a) or Ad-Null (b) group (*p *< 0.05). n = 2; 4 beads/mouse.

To confirm the proceeding finding that PEDF from Ad-PEDF gene transfer is associated with the reduction of tumor angiogenesis, and to directly demonstrate the causal relationship, we performed the alginate-encapsulated tumor cell assay, which is capable of demonstrating whether the development of tumor angiogenesis is prevented by PEDF treatment. As shown in Fig 6, the intensity of blood vessels on the surface of tumor cell-containing alginate beads was noticeably less in Ad-PEDF-treated mice than Ad-null or NS treated mice (Fig 6A). The quantification results for the amount of FITC-dextran indicate that the distribution of extravasated tracer in the encapsulated tumor tissues was consistent with the distribution of blood vessels on the bead surface; the amount of FITC-dextran per beads in Ad-PEDF, Ad-null and NS group was 2.1 ± 0.3 μg/bead, 5.8 ± 0.3 μg/bead and 6.2 ± 0.6 μg/bead, respectively. Similarly, an experiment with encapsulated B16-F10 cells has been performed and its result was similar to the result for CT-26 cells. (Data were not shown). These data confirmed that Ad-PEDF-mediated PEDF gene transfer and expression is responsible for the inhibition of tumor angiogenesis in the studied tumor model.

## Discussion

Angiogenesis is required for a variety of physiological and pathological processes. It is a complex biological process under precise regulation of multiple factors in multiple steps [19]. There are two groups of reciprocally antagonizing factors, proangiogenitors and antiangiogenitors. The former includes vascular endothelial growth factor (VEGF), platelet-derived growth factor (PDGF), basic fibroblast growth factor (b-FGF) and fibroblast growth factor (FGF), and the latter includes angiostatin, endostatin, thrombospondin and PEDF. PEDF is notable for its inhibitory function responsible for the avascularity of ocular compartments by opposing the angiogenic effect of VEGF in eye. It has been shown that PEDF is the most potent endogenous inhibitor of angiogenesis; its potency is twice that of angiostatin and seven times of endostatin [5,20]. Recently, data showed that PEDF also plays important roles in regulating normal development and tumor growth. For example, a recent study showed that the expression of PEDF is inversely proportional to the expression of VEGF at the growth plate of cartilage and is involved in the control of osteosarcoma [21]. In addition, it has been reported that PEDF could significantly inhibit neuroblastoma and Wilms' tumor [22,23]. A decrease of PEDF results in a tumor-permissive environment and promotes tumor growth and metastasis [9]. It is generally thought that PEDF's anti-tumor activity is the extended function of its antiangiogenic effect, decreasing microvascularity and blood supplying.

In the past decade, researchers have prepared various forms of PEDF and demonstrated its beneficial effects in several tumor models. Doll *et al *reported that exogenous recombinant PEDF protein induced tumor epithelial apoptosis in mouse prostate and pancreas [24]. Liu *et al *showed that a short peptide derived from the parent PEDF molecule was able to inhibit osteosarcoma growth [25]. Streck *et al *reported that adeno-associated virus (AAV) delivering PEDF gene treatment successfully restricted human neuroblastoma engraftment in a dose-dependent fashion [22]. Hase *et al *demonstrated that intratumoral injection of a lentivirus vector encoding PEDF resulted in inhibition of human pancreatic cancer in nude mice [20]. Moreover, Wang *et al *showed that *in vivo *transfer of PEDF mediated by adenoviral vectors exerted a dramatic inhibition of tumor growth in athymic nude mice implanted with the human HCC and in C57BL/6 mice implanted with mouse lung carcinoma [26]. In the present study, we investigated the adenovirus-mediated PEDF gene transfer and tested its anti-tumor effect in a mouse model of melanoma.

Melanoma, a tumor derived from neuroectoderm, has a high malignancy with poor prognosis, due to the vascular and lymphatic metastasis during the late stage [27]. The outcomes of existing therapeutic protocols are very poor. Thus, the development of novel treatment approaches is required [28]. Since the neovascularization is one critical underlying mechanism of vascular and lymphatic metastasis, the current study was designed to investigate whether overexpression of PEDF mediated by adenovirus gene transfer is a potential approach to suppress tumor angiogenesis and inhibit melanoma growth. Encouragingly, we constructed a recombinant PEDF adenovirus that is capable of transferring PEDF gene producing secretory PEDF protein both *in vitro *and *in vivo*. Furthermore, we showed that the secretory PEDF is a functional protein with potent inhibitory effects on HUVEC proliferation. More importantly, tumor-bearing mice exhibited significantly reduced tumor volume and prolonged survival time after Ad-PEDF treatment. Finally, we demonstrated that Ad-PEDF exerted anti-tumor activity through inhibiting angiogenesis, reducing MVD and increasing apoptosis.

Adenovirus type 5 is an established and widely used vector for the delivery of therapeutic genes [29]. Although there is no evidence to prove Ad-PEDF has a stronger therapeutic effect on tumors than other PEDF patterns, the adenovirus vector has several properties that make it particularly promising for gene therapy. First, the adenovirus vector can efficiently transfer genes to both dividing and quiescent cells both *in vivo *and *in vitro*, and importantly possesses high stability *in vivo*. Additionally, adenovirus vector can be produced at high titer conveniently, which is essential for clinical utility. Finally, as opposed to the retrovirus vector such as lentivirus, adenoviral DNA does not usually integrate into host cell's genome and therefore has a very low risk of generating tumorigenic mutations. Adenovirus-related pathology is mostly limited to mild upper respiratory tract infections [30,31].

It is very encouraging that Ad-PEDF treatment resulted in a high level of PEDF expression in serum and caused the inhibition of tumor growth. However, a few questions were left unaddressed in this study. First, this study mainly focused on the primary tumor, it is unknown whether Ad-PEDF treatment is effective in controlling late stage tumor growth, metastasis, and tumor growth in a metastasis site. Based on the assumption that Ad-PEDF may be mainly used as an option for advanced melanoma treatment, investigation of the effect of Ad-PEDF treatment for a late stage melanoma is critical to evaluate its potential clinical value, which is one of our future directions. In addition, adenovirus is highly immunogenic, which induces major humoral and cellular immune response when administered systemically [30]. These immune responses result in a quick clearance of virus when they are re-administered. While the adenovirus-induced humoral immune response leads to the antibody-mediated neutralization of virus in circulation, the cell-mediated immune response results in lysis of adenovirus-infected cells and loss of transferred gene. To prevent this quick clearance, we treated animals with multiple injections of Ad-PEDF every 3 days in this study. Although we used a lower dose than in the literature, the optimal window for effective dose and toxicity of this treatment is still to be determined. Furthermore, consistent with previous observation, Ad5, used in the present study was mainly directed to the liver, probably, via the vitamin K-dependent coagulation zymogens or other plasma protein-directed mechanisms [32]. We speculate that the secretory PEDF from non-tumor tissues is first released into the blood, then circulates to tumor tissue and exerts the antiangiogenesis effect. It appears not necessary to avoid the liver uptake of virus in our model and liver is probably the major source of the serum PEDF after Ad-PEDF treatment. However, because of the potential and undefined side effects and to further increase anti-tumor efficacy, modification of vector or optimization of delivery route to direct viruses into tumor tissue is critical to translate this study to an applicable therapeutic option for patients.

It has been shown that the liposome system can reduce adenoviral immunogenicity, increase localization of virus, and allow successful re-administration of the virus without loss of gene expression efficiency [16]. Therefore, we developed an Ad-PEDF-liposome system and are under active investigation, aiming to address the above mentioned unanswered issues. In addition, to further increase efficacy and limit side effects, we are also exploring the bi-specific antibody strategy to retarget the Ad-PEDF adenovirus to melanoma tumor tissue, as Reynolds *et al *prepared a targetable adenovirus-mediated gene transfer to pulmonary endothelium [33,34]

In summary, until the current study, research for experimental melanoma treated with Ad-PEDF had not been reported. Our data validate that Ad-PEDF treatment can exert an inhibitory effect on tumor angiogenesis. While the adenovirus-mediated PEDF gene therapy may provide a promising approach for primary melanoma treatment, we are still exploring the strategies for reducing its side effects and improving the tropism of Ad-PEDF to tumor.

## Conclusion

Our study indicates that adenovirus-mediated PEDF gene transfer and expression can provide an effective approach to inhibit primary mouse B16-F10 melanoma growth.

## Competing interests

The authors declare that they have no competing interests.

## Authors' contributions

LPY carried out transfection and viral preparation, animal experiment and histological analysis, and drafted the manuscript. PC carried out TUNEL staining and performed statistical analyses. XCP contributed to animal experiment and revised the manuscript. HSS, WHH, FYC and STL contributed to animal experiment. LY offered Adenovirus and designed the topic. YQW supervised experimental work and revised the manuscript. All authors read and approved the final manuscript.
